# Dynamic distribution and expression *in vivo *of the human interferon gamma gene delivered by adenoviral vector

**DOI:** 10.1186/1471-2407-9-55

**Published:** 2009-02-16

**Authors:** Jiangxue Wu, Xia Xiao, Hongyun Jia, Jiemin Chen, Yinghui Zhu, Peng Zhao, Huanxin Lin, Wenlin Huang

**Affiliations:** 1 State Key Laboratory of Oncology in South China, Cancer Center, Sun Yat-sen University, Guangzhou 510060, PR China; 2 Institute of Microbiology, Chinese Academy of Science, Beijing 100080, PR China; 3 College of fundamental Medical Science, Guangzhou University of Chinese Medicine, Guangzhou 510006, PR China

## Abstract

**Background:**

We previously found that r-hu-IFNγ exerts a potent anti-tumor effect on human nasopharyngeal carcinoma xenografts *in vivo*. Considering the fact that the clinical use of recombinant IFNγ is limited by its short half-life and systemic side effects, we developed a recombinant adenovirus, Ad-IFNγ.

**Methods:**

Dynamic distribution of the adenovirus vector and expression of IFNγ were evaluated by Q-PCR and ELISA after intratumoral administration of Ad-IFNγ into CNE-2 xenografts.

**Results:**

Ad-IFNγ DNA was mainly enriched in tumors where the Ad-IFNγ DNA was injected (*P *< 0.05, compared to blood or parenchymal organs), as well as in livers (*P *< 0.05). Concentrations of Ad-IFNγ DNA in other organs and blood were very low. Intratumoral Ad-IFNγ DNA decreased sharply at high concentrations (9 × 10^5 ^copies/μg tissue DNA), and slowly at lower concentrations (1.7–2.9 × 10^5 ^copies/μg tissue DNA). IFNγ was detected in the tumors and parenchymal organs. The concentration of IFNγ was highest in the tumor (*P *< 0.05), followed by the liver and kidney (*P *< 0.05). High-level intratumoral expression of IFNγ was maintained for at least 7 days, rapidly peaking on day 3 after injection of Ad-IFNγ DNA.

**Conclusion:**

An IFNγ gene delivered by an adenoviral vector achieved high and consistent intratumoral expression. Disseminated Ad-IFNγ DNA and the transgene product were mainly enriched in the liver.

## Background

IFNγ, a homodimeric cytokine produced by T lymphocytes and natural killer cells, belongs to a family of glycoproteins that have antiviral, immunomodulatory, and anti-proliferative effects. The anti-tumor activities of IFNγ have been shown in various kinds of tumors, such as lymphomas, melanomas, and metastatic renal cell carcinomas [1-4]. Nasopharyngeal carcinoma (NPC) is a malignant disease of the head/neck region that is endemic in Southeast Asia [5]. We have reported that r-hu-IFNγ treatment exerts anti-proliferative effects *in vitro *on NPC cells, and leads to a profound anti-tumor effect *in vivo *[6]. However, although IFNγ has shown some promise in preclinical and clinical anticancer studies, it possesses properties that limit its clinical use.

High serum concentrations of IFNγ yield significant side effects and toxicities. These side effects include fever, fatigue, nausea, vomiting, neurotoxicity, and leukopenia. In addition, IFNγ has a short half-life of elimination, whether given intravenously, subcutaneously, or intramuscularly, and thus requires relatively frequent readministration. These limitations have prompted the investigation of alternate modes of delivering this cytokine to achieve better therapeutic outcomes while minimizing toxicity [1]. Studies have revealed that the most efficient and least toxic delivery system for IFNγ is a localized, sustained release dosage form [1,3,4]. Therefore, gene therapy with intratumorally injected recombinant adenoviral vectors is a promising approach because it offers the potential to achieve consistent therapeutic gene expression in the tumor [3,4].

In the present study, we developed a recombinant adenovirus, Ad-IFNγ, encoding human interferon gamma. To determine whether intratumoral administration of Ad-IFNγ could achieve localized sustained expression of IFNγ, we sought to characterize the biodistribution of the adenoviral vector and transgene expression in the tumor, blood, liver, kidney, heart, brain, spleen, and lung at days 1, 2, 3, 5, 7, 14, and 21 after injection. Results indicated that the IFNγ gene delivered by adenoviral vector achieved high intratumoral expression, lasting for at least 7 days after injection of Ad-IFNγ. Disseminated Ad-IFNγ DNA and the transgene product were mainly enriched in the liver.

## Methods

### Adenoviral vectors

E1-deleted, replication-deficient adenoviral vectors based on human adenovirus serotype Ad5 were used to produce the non-replicating adenovirus, Ad-IFNγ, encoding human interferon gamma [7]. The cDNA for IFNγ was inserted into the E1 region of the adenoviral genome with transgene expression driven by the cytomegalovirus (CMV) promoter. Adenoviruses were propagated in 293 cells (American Type Culture Collection, Manassas, VA, USA), harvested at 48 h after infection, and purified by cesium chloride gradient centrifugation according to a standard protocol [8], resulting in a particle:pfu ratio of 100:3.32. The viral vectors were stored at -80°C in virus preservation solution (10% glycerol, 0.585% NaCl, 0.02% MgCl_2_·H_2_O, 0.01 M Tris-HCl, pH 8.4) at a concentration of 1 × 10^12 ^VP/ml.

### Animals

Female BALB/c nude mice (4–6 weeks old) were obtained from the Experimental Animal Center (Sun Yat-sen University, Guangzhou, China) and maintained in a specific pathogen-free (SPF) environment. After 1 week of adaptation, mice were inoculated subcutaneously in the scapular region with 2 × 10^6 ^CNE-2 cells to generate tumors for subsequent experiments. When tumors reached 5–8 mm in diameter, mice were randomly assigned to groups (3 mice for each time point). Ad-IFNγ (diluted with 0.9% NaCl to 100 μl) or vehicle was delivered into the center of the tumor via a single injection with a 30-gauge needle. The dose used per injection was 1 × 10^10 ^VP/tumor. Samples of blood, tumor, heart, liver, spleen, lung, kidney, and brain were harvested at the indicated time points after virus injection, and stored in aliquots at -80°C for analysis. All the animal experiments were conducted in accordance with "Guidelines for the Welfare of Animals in Experimental Neoplasia".

### Quantitative analysis of adenovirus distribution

Tissue samples (about 50 mg for tissue) were immersed in liquid nitrogen and ground into fine tissue powder in a mortar. Viral DNA was isolated from the tissue powder and whole blood (about 100 μl for blood) using a Total DNA Extraction Kit (TIANGEN, Beijing, China).

### Quantitative PCR

Adenovirus copies in the samples were measured by hexon DNA PCR [9]. Primer and probe sequences were: hexon forward primer: 5'-CTTCGATGATGCCGCAGTG-3'; hexon reverse primer: 5'-GGGCTCAGGTACTCCGAGG-3'; hexon probe: 5'-FAM-TTACATGCACATCTCGGGCCAGGAC-TAMRA-3'. Amplification was performed in a 50 μl reaction volume under the following conditions: 10 ng of sample DNA, 1× Taqman Universal PCR Master Mix, 600 nM forward primer, 900 nM reverse primer, and 100 nM hexon probe. Thermal cycling conditions were: 2 min of incubation at 50°C, 10 min at 95°C, followed by 35 cycles of successive incubations at 95°C for 15 sec and 60°C for 1 min [9]. Quantification of adenovirus copy number was performed using a standard curve consisting of dilutions of adenovirus DNA from 1,500,000 to 15 copies in 10 ng of cellular genomic DNA.

### Quantitative analysis of IFNγ distribution

After being immersed in liquid nitrogen and ground into fine tissue powder in a mortar, tissue samples (about 50 mg) were ground with PBS containing Protease Inhibitor Cocktail II (Upstate, Lake Placid, NY, USA). After centrifugation, the supernatants were extracted and stored at -80°C. IFNγ concentrations in the tumor, organ extracts, and plasma samples (about 100 μl) were detected with a human IFNγ ELISA kit (R&D Systems, Minneapolis, MN, USA) according to the manufacturer's recommendations. The results were then converted to the concentrations of IFNγ in the different tissues based on the sample volume or weight. Sensitivity of the kit was up to 16 pg/ml.

### Statistical Analysis

Results were evaluated using a *t*-test by SPSS 11.0 software (SSPS Inc., Chicago, IL, USA). A *P *value < 0.05 was considered statistically significant.

## Results

### Systemic distribution of recombinant human IFNγ adenovirus

1 × 10^10 ^VP of Ad-IFNγ was injected into CNE-2 tumors. At the indicated time points post-injection, samples of tumor, parenchymal organs, and blood were harvested. To demonstrate adenovirus distribution, fluorescent real-time quantitative PCR was used to quantify viral copies in the samples.

**Figure 1:**
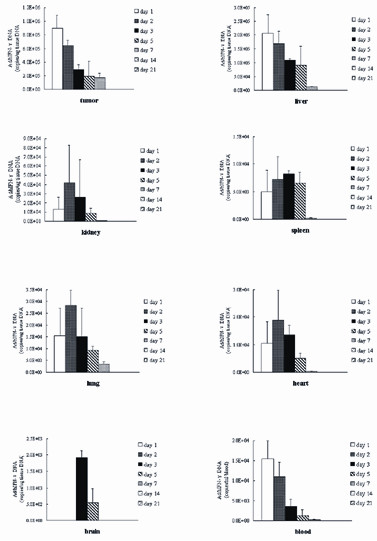
**Ad-IFNγ concentrations in the tumor, heart, liver, spleen, lung, kidney, brain, and blood at different time points after intratumoral injection of Ad-IFNγ**. The dose of injection was 1 × 10^10 ^VP/tumor. Data represent mean ± SD of three mice. Representative results from two independent experiments are shown.

Samples from tumor, heart, liver, spleen, lung, kidney, brain, and blood on days 1, 2, 3, 5, 7, 14 and 21, were tested for the amount of targeted DNA. The copy number of the targeted DNA in samples was deduced according to the standard curves. As shown in Fig. 1, Ad-IFNγ DNA was detected in tumor, blood, and parenchymal organs. Ad-IFNγ DNA was mainly enriched in tumors where the Ad-IFNγ DNA was injected (*P *< 0.05, compared to blood or parenchymal organs), then in livers (*P *< 0.05, compared to blood or other parenchymal organs). Concentrations of Ad-IFNγ DNA were very low in other organs and blood (Fig. 1). Ad-IFNγ DNA persisted for at least 7 days in the tumors. Intratumoral Ad-IFNγ DNA decreased sharply at high concentrations (9 × 10^5 ^copies/μg tissue DNA), and slowly at lower concentrations (1.7–2.9 × 10^5 ^copies/μg tissue DNA) (Table 1). Ad-IFNγ DNA in livers also decreased in a time-dependent manner and became undetectable at day 14 after intratumoral administration (Table 1).

**Table 1:** Adenovirus copies in tumors and livers were measured by hexon DNA PCR.

	tumor (copies/μg tissue DNA)	liver (copies/μg tissue DNA)
day 1	897927.2 ± 188201.2	206274.7 ± 67269.5
day 2	645995.1 ± 76137.4	168531.6 ± 46526.4
day 3	289441.2 ± 75017.3	109217.2 ± 5478.3
day 5	197677.1 ± 220320.1	90712.8 ± 68176.5
day 7	169882.6 ± 69067.4	11465.8 ± 2383.5
day 14	0	0
day 21	0	0

The dose of injection was 1 × 10^10 ^VP/tumor. Data represent mean ± SD of three mice. Representative results from two independent experiments are shown.

### Systemic distribution of human IFNγ expressed by Ad-IFNγ

**Figure 2:**
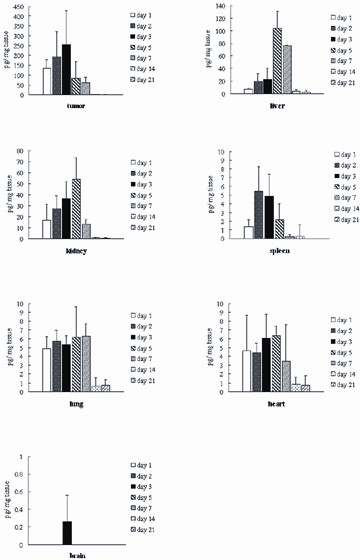
**Human IFNγ concentrations in the tumor, heart, liver, spleen, lung, kidney, and brain at different time points after intratumoral injection of Ad-IFNγ**. The dose of injection was 1 × 10^10 ^VP/tumor. Data represent mean ± SD of three mice. Representative results from two independent experiments are shown.

To evaluate the dynamic expression *in vivo *of the transgene from the recombinant human IFNγ adenovirus, we used ELISA to test the levels of IFNγ in samples collected from tumor, heart, liver, spleen, lung, kidney, brain, and blood on days 1, 2, 3, 5, 7, 14, and 21 post-injection of Ad-IFNγ. IFNγ was detected in tumor and parenchymal organs, while IFNγ was undetectable in blood (Fig. 2). The concentration of IFNγ in tumor was highest (*P *< 0.05, compared to blood or parenchymal organs), then in liver and kidney (*P *< 0.05, compared to blood or other parenchymal organs). As shown in Fig. 2, intratumoral concentrations of IFNγ protein, in contrast to the amount of Ad-IFNγ DNA, rose rapidly and peaked on day 3 after injection of Ad-IFNγ DNA, then decreased sharply. On day 14 and 21, only a minimal amount of IFNγ remained in the tumor. Concentrations of IFNγ in the liver and kidney peaked on day 5. On day 7, IFNγ in all tissues decreased dramatically with the exception of maintained high-level expression in the liver. While low levels of IFNγ were still present in the liver on days 14 and 21, IFNγ in other organs was almost undetectable.

## Discussion

Nasopharyngeal carcinoma is a major malignant disease of the head and neck region that is endemic to Southeast Asia and the Mediterranean basin. NPC affects a predominantly young population and the current treatment regimen of radiation therapy, even combined with cisplatin chemotherapy, yields a 5-year survival rate of approximately 70%. Therefore, evaluation and development of novel therapeutic approaches are critical [9-14]. We had previously found that r-hu-IFNγ has direct anti-proliferative effects on all NPC cell lines tested (CNE-1, CNE-2 and C666-1) [6]. Moreover, r-hu-IFNγ potently inhibits the growth of CNE-2 and C666-1 xenografts *in vivo *[6]. Thus, our study revealed that r-hu-IFNγ treatment is a promising novel approach for the treatment of NPC. However, the clinical application of recombinant IFNγ is limited by the short half-life and the systemic side effects experienced by patients. Therefore, continuous localized release of IFNγ is a desirable goal. To overcome these difficulties, we developed a recombinant adenovirus, Ad-IFNγ. Our results suggested that high and sustained expression of IFNγ could be achieved in CNE-2 xenografts, after intratumoral injection of Ad-IFNγ (Fig. 2). Consistent with the anti-tumor effects of r-hu-IFNγ, Ad-IFNγ produced a significant dose-dependent inhibition of tumor growth in CNE-2 models. Intratumoral administration of Ad-IFNγ at doses of 1 × 109 and 5 × 108 pfu/week for three weeks led to inhibition rates of 67.9% and 58.64%, respectively [see Additional file 1, Zhu YH, et al., unpublished data].

Intratumoral injection is an advantageous method for local viral gene delivery [15,16]. Researchers often assume that viral vectors and transgene expression are confined to solid tumors after the infusion, thereby causing minimal toxicity in normal tissues. However, the dissemination of the viral vector and its encoded gene product could be a serious impediment for vectors that encode cytokine genes, such as IFNγ, that are not only expressed *in situ*, but also secreted into the system and have considerable normal tissue toxicity [17-20]. Therefore, we investigated transgene expression in the blood and major organs in this study.

We found that there was potential dissemination of viral DNA and transgene product into parenchymal organs. A significant amount of IFNγ was detected in the liver and kidney, which indicated that the clearance of IFNγ *in vivo *is through hepatic metabolism and glomerular filtration. Our results also suggested that most of the disseminated viral DNA was enriched in the liver, which is consistent with reports of efficient uptake of adenovirus by the liver. There was a minimal amount of viral DNA in the blood. One possible explanation for this is that the number of infectious viruses present in the blood was significantly reduced through rapid uptake of the virus by Kupffer cells in the liver [21,22].

Studies have revealed that dissemination of transgene products in normal organs, such as in the liver, may exceed normal tissue tolerance if the products are highly toxic [19]. Toxicological studies conducted in this lab have indicated that intratumoral injection of Ad-IFNγ doesn't induce significant toxic effects in mice [unpublished data]. Therefore, adenovirus-mediated intratumoral delivery of the IFNγ gene is a promising approach because it achieves high and sustained local IFNγ expression in the tumor.

## Conclusion

Adenovirus-mediated intratumoral delivery of the IFNγ gene is a promising approach because it achieves high and sustained local IFNγ expression in the tumor. Disseminated Ad-IFNγ DNA and the transgene product were mainly enriched in the liver.

## Competing interests

The authors declare that they have no competing interests.

## Authors' contributions

JXW, XX, HYJ, JMC, YHZ, PZ and HXL participated in data acquisition. WLH supervised and participated with JXW in the design and coordination of the study. JXW drafted and wrote the manuscript. All authors read and approved the final manuscript.

## Pre-publication history

The pre-publication history for this paper can be accessed here:

http://www.biomedcentral.com/1471-2407/9/55/prepub

## Supplementary Material

Additional file 1 **Antitumor activity of Ad-IFNγ on CNE-2 xenografts.** The data provided represent the antitumor activity of Ad-IFNγ on CNE-2 xenografts. Female athymic nude mice were inoculated s.c. in the scapular region with 2 × 10^6 ^CNE-2 cells in 100 μl sterile PBS. When tumors reached a volume of 30 to 40 mm^3^, animals were randomly assigned into 6 experimental groups of 6–7 animals: Ad-IFNγ (1 × 10^9^, 5 × 10^8 ^or 1 × 10^8 ^pfu/week), 1 × 10^9 ^pfu/week of Ad-LacZ, 1 × 10^6 ^IU/kg/d of r-hu-IFNγ or PBS alone was intratumorally injected. Mice were killed after 3 weeks of treatment and tumors were resected and weighted. Columns, average weight of tumor from 6–7 mice; bars, SD. *, *p *< 0.05, compared with the PBS-treated and the Ad-LacZ group. Click here for file
