# Supplementary figures and images for: Dynamic distribution and expression in vivo of the human interferon gamma gene delivered by adenoviral vector
Source: BMC Cancer. 2009 Feb 16;9:55. doi: 10.1186/1471-2407-9-55 (PMC2667533; doi:10.1186/1471-2407-9-55)

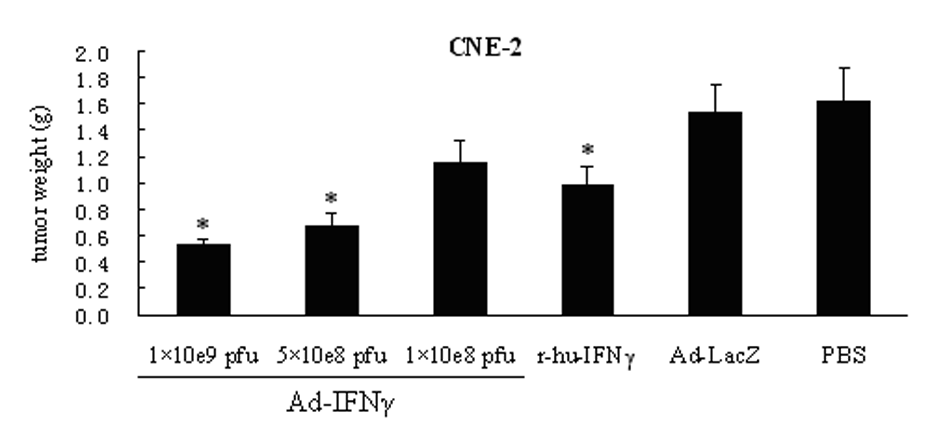

Supplement: Additional file 1 — Antitumor activity of Ad-IFNγ on CNE-2 xenografts. The data provided represent the antitumor activity of Ad-IFNγ on CNE-2 xenografts. Female athymic nude mice were inoculated s.c. in the scapular region with 2 × 10^6^ CNE-2 cells in 100 μl sterile PBS. When tumors reached a volume of 30 to 40 mm3, animals were randomly assigned into 6 experimental groups of 6–7 animals: Ad-IFNγ (1 × 10^9^, 5 × 10^8^ or 1 × 10^8^ pfu/week), 1 × 10^9^ pfu/week of Ad-LacZ, 1 × 10^6^ IU/kg/d of r-hu-IFNγ or PBS alone was intratumorally injected. Mice were killed after 3 weeks of treatment and tumors were resected and weighted. Columns, average weight of tumor from 6–7 mice; bars, SD. *, p < 0.05, compared with the PBS-treated and the Ad-LacZ group. [file 1471-2407-9-55-S1.tiff]
